# The other-race effect on the McGurk effect in infancy

**DOI:** 10.3758/s13414-021-02342-w

**Published:** 2021-08-13

**Authors:** Yuta Ujiie, So Kanazawa, Masami K. Yamaguchi

**Affiliations:** 1 Graduate School of Psychology, Chukyo University, 101-2 Yagoto Honmachi, Showa-ku, Nagoya-shi, Aichi 466-8666 Japan; 2 Japan Society for the Promotion of Science, 5-3-1 Kojimachi, Chiyoda-ku, Tokyo, 102-0083 Japan; 3 Research and Development Initiative, Chuo University, 1-13-27 Kasuga, Bunkyo-ku, Tokyo, 112-8551 Japan; 4 Research Organization of Open Innovation and Collaboration, Ritsumeikan University, 2-150 Iwakura-cho, Ibaraki, Osaka 567-8570 Japan; 5 Department of Psychology, Japan Women’s University, 2-8-1 Mejirodai, Bunkyo-ku, Tokyo, 112-8681 Japan; 6 Department of Psychology, Chuo University, 742-1 Higashi-Nakano, Hachioji, Tokyo, 192-0393 Japan

**Keywords:** Development, Multisensory processing

## Abstract

This study investigated the difference in the McGurk effect between own-race-face and other-race-face stimuli among Japanese infants from 5 to 9 months of age. The McGurk effect results from infants using information from a speaker’s face in audiovisual speech integration. We hypothesized that the McGurk effect varies with the speaker’s race because of the other-race effect, which indicates an advantage for own-race faces in our face processing system. Experiment 1 demonstrated the other-race effect on audiovisual speech integration such that the infants ages 5–6 months and 8–9 months are likely to perceive the McGurk effect when observing an own-race-face speaker, but not when observing an other-race-face speaker. Experiment 2 found the other-race effect on audiovisual speech integration regardless of irrelevant speech identity cues. Experiment 3 confirmed the infants’ ability to differentiate two auditory syllables. These results showed that infants are likely to integrate voice with an own-race-face, but not with an other-race-face. This implies the role of experiences with own-race-faces in the development of audiovisual speech integration. Our findings also contribute to the discussion of whether perceptual narrowing is a modality-general, pan-sensory process.

This study aimed to investigate the difference in the McGurk effect between the own-race-face and other-race-face stimuli among Japanese infants from 5 to 9 months of age. The McGurk effect is a well-known illusion demonstrating the role of seeing a face in audiovisual speech integration (e.g., McGurk & MacDonald, 1976). An example of this effect is that when a movie of a mouth articulating the phoneme sequence /ka/ is dubbed with a voice uttering a different initial phoneme /pa/, these are integrated to cause a fused percept of an intermediate phoneme in /ta/. A manifestation of the McGurk effect is involved in the development of sensitivity to audiovisual correspondences in speech (e.g., Rosenblum, 2008; Rosenblum et al., 1997). In infant development, the McGurk effect has been observed from the preverbal stage (Desjardins & Werker, 2004; Rosenblum et al., 1997). By 4 months, infants learn the relation between auditory speech and facial speech information (Kuhl & Meltzoff, 1982, 1984; Patterson & Werker, 1999, 2002). At around 5 months, infants can integrate a voice with an incongruent facial speech pattern and perceive the McGurk effect in a certain syllable combination (Desjardins & Werker, 2004; Rosenblum et al., 1997). Rosenblum et al. (1997) habituated 5-month-old infants with the speech of auditory /va/ with visual /va/, then presented them with two test stimuli, auditory /ba/ with visual /va/, which causes the McGurk effect (/va/), and auditory /da/ with visual /va/, which is perceived as /da/. The results revealed that the infants showed dishabituation to the stimulus of auditory /da/ with visual /va/, showing that the infants could integrate auditory /ba/ with visual /va/ and perceive the McGurk effect (/va/) as adults did.

We would like to propose the possibility that the McGurk effect in infants should vary with the speaker’s race because of the other-race effect in our face-processing system (e.g., Nelson, 2001; Valentine, 1991). The other-race effect indicates an advantage for own-race faces in our face processing system—that is, we are good at recognizing similar-race faces that we see most often but not other-race faces that we rarely see (e.g., Bothwell et al., 1989; MacLin & Malpass, 2001; Malpass & Kravitz, 1969; Valentine, 1991). This effect is considered to come from the different amount of experience with own-race and with other-race individuals during infancy (Anzures et al., 2012; Bar-Haim et al., 2006; Kelly et al., 2007; Kelly et al., 2005,). Kelly et al. (2007) assessed the ability to discriminate faces of own race and other races (African, Middle Eastern, and Chinese) in 3- to 9-month-old Caucasian infants who frequently experienced Caucasian faces from birth. Results showed that 3-month-old infants could recognize faces of all races, but 6-month-old infants were able to recognize Caucasian and Chinese faces only, and the 9-month-old infants were able to recognize only own-race faces. Such a developmental process was replicated in populations of Asian infants (Kelly et al., 2009). These results show that the other-race effect emerges at 6 months and is fully present at 9 months (e.g., Kelly et al., 2005; Kelly et al., 2007), implying that the onset of the other-race effect in infancy is close to the age at which infants can perceive the McGurk effect (e.g., Rosenblum et al., 1997). The current study examined the hypothesis of whether the other-race effect appears in processing facial speech in the context of McGurk effect.

Such a manifestation of the other-race effect in the McGurk effect can contribute to the discussion of whether perceptual narrowing is a modality-general, pan-sensory process (e.g., Lewkowicz & Ghazanfar, 2009; Pascalis et al., 2014; Pons et al., 2009). Perceptual narrowing shows the developmental process in order to attune to the environment during infancy (e.g., Maurer & Werker, 2014; Pascalis et al., 2002). In the development of speech processing, younger infants can discriminate phonetic contrasts regardless of language, but this ability narrows to the discrimination of phonetic contrasts that are native to their own language in older infants, similar to that in adults (e.g., Kuhl et al., 1992; Werker & Tees, 1983). Likewise, in the development of face processing, the ability to discriminate faces works more broadly in younger infants, but is gradually attuned to own-race faces that we see most often from birth (e.g., Kelly et al., 2005; Kelly et al., 2007; Nelson, 2001; Pascalis et al., 2002; Quinn et al., 2016). A recent focus of the discussion of perceptual narrowing is whether experiences with both native languages and specific faces play a role in the development of audiovisual speech perception, which is the hypothesis of the modality-general, pan-sensory narrowing process (e.g., Lewkowicz & Ghazanfar, 2009; Pons et al., 2009). Indeed, the role of experience with native language has been found in audiovisual speech perception (e.g., Kubicek, de Boisferon, et al., 2014a; Kubicek, Gervain, et al., 2014b; Pons et al., 2009), as well as in auditory speech perception (e.g., Kuhl, 1991; Werker & Tees, 1983, 2002). On the other hand, the role of experience with own-race faces in the development of audiovisual speech perception remains unclear. Demonstrating an other-race effect on the McGurk effect would support the hypothesis of a pan-sensory narrowing process by experience with specific faces.

In summary, this study aimed to find the other-race effect on the McGurk effect among Japanese 5–6-month-old and 8–9-month-old infants. We hypothesized that infants are likely to integrate voice with an own-race face that they see often but not with an other-race face that they rarely experience. To test this, we conducted three experiments using a familiarization/novelty preference method. In Experiment 1, we tested whether the infants are likely to perceive the McGurk effect by an own-race-face speaker but not by an other-race-face speaker. In Experiment 2, we attempted to replicate *the other-race effect* on the McGurk effect, by manipulating combinations of face-voice identity cues. In Experiment 3, as a control experiment, we confirmed whether the infants can differentiate two auditory syllables (/pa/ and /ka/).

## Experiment 1

In this experiment, we examined whether infants are likely to integrate audiovisual speech of an own-race-face speaker but not of an other-race-face speaker using a familiarization/novelty preference method. We established two race-face conditions, each of which consisted of two phases, the familiarization phase and the test phase. In the familiarization phase, we presented infants with six familiarization trials, which repeated the McGurk stimulus (auditory /pa/ and visual /ka/) six times per trial. In the test phase, we presented the infants with the familiarized trial (auditory /ta/ with vegetable images, six times) and the novel trial (auditory /pa/ with vegetable images, six times). We expected that if infants can perceive the McGurk effect, they would become familiarized with the “/ta/” sound in the familiarization phase, and thus they would look longer at the novel trial (/pa/) in the test phase. In our hypothesis, a significant preference for the novel trial in the test phase would result from the presence of audiovisual speech integration in the familiarization phase.

### Methods

#### Participants

Thirty-two infants ages 5–6 months (12 girls and 20 boys; mean age = 175 days, range: 148 to 192 days) and 32 infants ages 8–9 months (15 girls and 17 boys; mean age = 254 days, range: 225 to 294 days) participated in Experiment 1, all of whom were raised by their Japanese parents. All infants were full term at birth and were healthy at the time of the experiment. An additional 17 infants were excluded due to fussiness (*n* = 3) or failure to be familiarized to the speech movie (*n* = 14). This experiment was conducted according to the Declaration of Helsinki and was approved by the Ethical Committee of Chuo University. The parents provided written informed consent for their infant’s participation prior to the study.

#### Stimuli

For the stimuli, we created the McGurk stimuli (auditory /pa/ and visual /ka/) and auditory stimuli (/pa/ and /ta/) from recordings of two females’ utterances for three syllables (/pa/, /ta/, and /ka/). We created our stimuli by referring to previous studies, where the stimuli comprised infant-directed speech of vowels and lasted around 3 seconds per utterance (Altvater-Mackensen & Grossmann, 2016, 2018). The speakers were one East Asian (native Japanese speaker, 22 years old) and one Caucasian (native English speaker, 23 years old) female. In order to make the speech stimuli prosodically similar between English and Japanese speakers, we recorded infant-directed speech (IDS), which has been shown to be relatively similar regardless of the language (e.g., Piazza et al., 2017). The visual stimuli (800 × 450 pixels) were recordings of the speakers’ faces, made using a digital video camera (GZ-EX370; JVC Kenwood, Yokohama, Japan). The voices (digitized at 48 kHz with a 16-bit quantization resolution) were recorded using a dynamic microphone (MD42; Sennheiser, Wedemark, Germany). For the McGurk stimuli, we used Adobe Premiere Pro CS6 (Adobe Systems, San Jose, CA, USA) to combine the /pa/ voice with the facial movement for /ka/ by adjusting the onset of voice (/pa/) based on the onset of the original utterance (/ka/). Pink noise was added to the voices (the signal-to-noise ratio was 0 dB) to induce perception of the McGurk effect (e.g., Sekiyama & Tohkura, 1991; Ujiie et al., 2015). Finally, there was one McGurk stimulus (auditory /pa/ and visual /ka/) for familiarization and two auditory stimuli (/pa/ and /ta/) for the test, for each speaker. In all of our stimuli, the sounds started 500 ms after the video appeared. The utterance then lasted around 1,700 ms and ended with the mouth completely shut in a neutral position at around 2,200 ms after stimulus onset. The duration of each stimulus was 2.8 s.

#### Apparatus

The visual stimuli were displayed on a 21-inch CRT monitor with a resolution of 1,024 × 768 pixels. The monitor was placed in front of the infant at a distance of 40 cm. The audio stimuli were presented at a sound pressure level of approximately 60 dB through two loudspeakers located on the left and right sides of the display. To record the infant’s looking behavior, a pinhole camera was set below the display.

#### Procedure

**Fig. 1:**
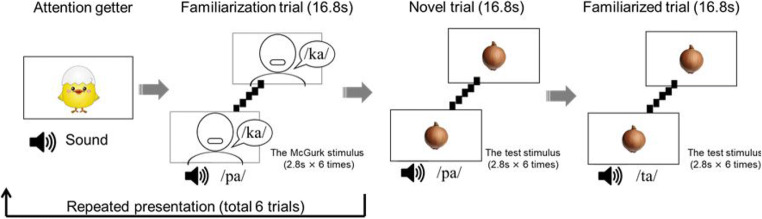
An example of the stimulus procedure. In the test phase, the order of presentation of the test trials (novel and familiarized) was randomly counterbalanced across the infants

We used the familiarization/novelty preference method to test whether infants are more likely to integrate audiovisual speech of an own-race-face speaker but not of an other-race-face speaker. We set two conditions of speakers’ faces: the own-race-face (East Asian) and the other-race-face (Caucasian). Each condition consisted of a familiarization phase and a test phase. An example of the stimulus procedure is shown in Fig. 1. The familiarization phase consisted of six trials, each of which consisted of the six-times-repeated presentation of the McGurk stimulus. The presentation of an animated audiovisual attention-getter in the center of the screen preceded each trial. In order to ensure that all infants began to familiarize with the stimuli from the same location, the next trial began only when the infant looked at the fixation point. The test phase consisted of two trials, a familiarized trial and a novel trial. In the familiarized trial, an auditory “/ta/” was presented with images of vegetables six times. In the novel trial, an auditory “/pa/” was presented with images of vegetables six times. Each trial lasted 16.8 s and was preceded by the presentation of the fixation cue in the center of the monitor. The order of presentation of the two trials was randomly counterbalanced across the infants. In our paradigm, we assumed that, the infants were familiarized with the percept (/ta/) in the familiarization phase if they perceived the McGurk effect, and then showed a novelty preference for the auditory syllable (/pa/) in the test phase.

Half of the infants, 16 infants from each age group, were assigned to the own-race-face condition, while the other half of the infants were assigned to the other-race-face condition. An appropriate sample size (*n* ≥ 16) for each age group was calculated via a power analysis (pwr package for R Version 3.3.0) to detect a Cohen’s *d* of 1.0, a statistical power (β) of .80, and a significance level (α) of .05 in a two-tailed one-sample *t* test against chance level. Each infant was seated on her (or his) parent’s lap. The viewing distance was approximately 40 cm. The infants looked at the stimuli on the monitor without any active task. Their behavior was recorded digitally throughout the experiment.

#### Data analysis

The observer measured the infant’s looking time based on an offline video movie. The observer, blind to the presentation order of the test phase, recorded the infant’s looking time by pressing a key while the infant was looking at the display. When the infant looked away from the presentation field, the key was lifted. In the following analysis, we excluded infants whose mean looking times in the last three trials were longer than in the first three trials during the familiarization phase (e.g., Ujiie et al., 2015). Those infants were deemed to not be sufficiently familiarized with the stimuli. Interobserver reliability was calculated based on the correlation between the looking times rated by the two observers across all conditions. The Pearson correlation between the two observers’ ratios demonstrated that the rating reached a sufficiently reliable level (*r* = .93).

### Results

#### Familiarization trials

The mean total looking time across the first half and second half of the familiarization trials in the own-race-face condition and the other-race-face condition are summarized in Table 1. To test whether an infant’s looking time during the familiarization trials differed between the two race-face conditions, we conducted a mixed analysis of variance (ANOVA), with trials (the first half and the second half of the familiarization phase) as a within-participants factor and the familiarization condition (the own-race-face and the other-race-face) as a between-participants factor, separately for each age group. In 5–6-month-olds, a mixed ANOVA showed a significant main effect of trials, *F*(1, 30) = 35.19, *p* < .01, η^2^ = .20. The main effect of the stimulus, *F*(1, 30) = 1.44, *ns,* and an interaction effect, *F* (1, 30) = 2.38, *ns,* were not significant. In 8–9-month-olds, the main effect of the trials was significant, *F*(1, 30) = 50.69, *p* < .01, η^2^ = .20, while the main effect of the stimulus, *F*(1, 30) = .01, *ns,* and an interaction effect, *F*(1, 30) = 2.18, *ns,* were not significant. We also calculated the difference of mean looking times between the first half and the second half of the familiarization trials and compared them between two race conditions utilizing a two-sample *t* test. The *t* tests showed no significant difference in 5- to 6- month-olds infants (*t* = 1.54, *df* = 30, *ns*) and 8- to 9-month-olds infants (*t* = .78, *df* = 30, *ns*). These results indicate that all infants in both age groups were familiarized to the McGurk stimuli without any differences in looking time during the familiarization phase between the own-race-face condition and the other-race-face condition.

**Table 1:**
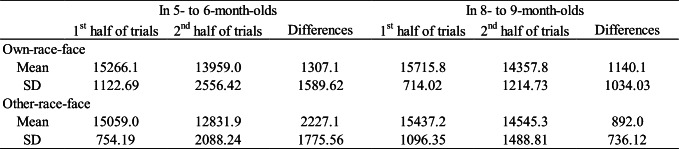
Mean total looking times (ms) across the first half and second half of the familiarization trials in both the own-race-face condition and the other-race-face condition

#### Test trials

**Fig. 2:**
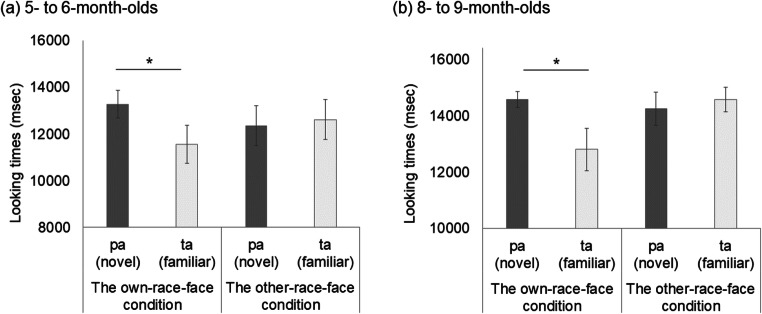
Mean total fixation times during the test phase of two familiarization conditions: in 5–6-month-olds (**a**) and in 8–9-month-olds (**b**). Dark-gray bars represent the results for the novel trial (the presentation of auditory /pa/ with vegetable images), and light-gray bars indicate the results for the familiarized trials (the presentation of auditory /ta/ with vegetable images). The error bars represent ± 1 standard error of the mean. Asterisks indicate the significance level of the statistical differences: *p < .05

The mean total looking times during the test phase in both the own-race-face condition and other-race-face condition are summarized in Fig. 2. We conducted a mixed ANOVA separately for each age group, with trial as a within-participants factor and the familiarization condition as a between-participants factor. In both age groups, the main effect of the stimulus was not significant, in 5–6-month-olds, *F*(1, 30) = .00, *ns*; in 8–9-month-olds, *F*(1, 30) = 1.55, *ns*), which indicates no difference in total looking time across the test phase between the own-race-face condition and the other-race-face condition. A Trials × Stimulus interaction was significant in 5–6-month-olds, *F*(1, 30) = 5.35, *p* < .05, η^2^ = .03, as well as in 8–9-month-olds, *F*(1, 30) = 4.56, *p* < .05, η^2^ = .06.

The novelty preference was evaluated with multiple *t* tests (corrected using the Holm method) within each age group to compare the mean total looking time between the familiarized and novel trials in both race-face conditions. In 5–6-month-olds, a significant preference was shown for the novel trial in the own-race-face condition, *t*(15) = 2.73, *p* = .02, *d* = .61, but not in the other-race-face condition, *t*(15) = .45, *ns*. Likewise, 8–9-month-old infants showed a significant preference for the novel trial in the own-race-face condition, *t*(15) = 2.70, *p* = .02, *d* = .79, but not in the other-race-face condition, *t*(15) = .45, *ns*. These results indicate that infants in both age groups perceived the McGurk effect (/ta/) during the familiarization phase in the own-race-face condition, but not in the other-race-face condition.

### Discussion

This experiment aimed to test whether infants are more likely to integrate auditory and visual speech information of an own-race speaker compared to an other-race speaker. Our results showed that infants in both age groups showed the novelty preference for the /pa/ trial in the own-race-face condition, but not in the other-race-face condition. These results indicate that the infants perceive the McGurk effect only when a speaker’s face was the own-race that the infants frequently experienced.

We also found that the onset of the other-race effect on the McGurk effect appears by 5–6-month-olds. This was not surprising because infants begin to show the sensitivity to ethnic differences of faces from 3 months (Kelly et al., 2005). Moreover, the reduced sensitivity to other-race-faces emerges at around 6 months although the other-race effect in face perception was observed stably and clearly at the age of 8 months (e.g., Kelly et al., 2007; Kelly et al., 2009). Besides, at around 6 months, infants can automatically use facial speech in audiovisual speech integration, as can adults (e.g., Rosenblum et al., 1997). Therefore, we considered that the decline in sensitivity to other-race-face in 5–6-month-olds was not contradictory to previous results (e.g., Kelly et al., 2007; Kelly et al., 2009).

In the next experiment, we attempted to show the robustness of the other-race effect on the McGurk effect. To confirm this, we manipulated the coherence of speech identity cues by swapping face and voice between stimuli (Green & Kuhl, 1991). Although infant-directed speech makes the stimuli prosodically similar (e.g., Piazza et al., 2017), there would be a difference in accents between the own-race (Japanese) and other-race (English) speakers. To claim the other-race effect on the McGurk effect, we needed to eliminate the possibility that the other-race speaker’s accent disrupted the perception of the McGurk effect. To test this, we manipulated the McGurk stimuli in the next experiment; the own-race-face stimulus was dubbed onto the other-race-speaker’s voice, while the other-race-face stimulus was dubbed onto the own-race-speaker’s voice. If the factor of a speaker’s race plays an important role in the McGurk effect, we expected to replicate the results of Experiment 1. That is, infants would perceive the McGurk effect by the own-race face stimuli but not by the other-race stimuli, regardless of the difference between the own-race and the other-race speaker’s accent.

## Experiment 2

### Methods

#### Participants

Another 32 infants ages 5–6 months (18 girls and 14 boys; mean age = 175 days, range: 141 to 194 days) and 32 infants ages 8–9 months (14 girls and 18 boys; mean age = 261 days, range: 242 to 282 days) participated in Experiment 2, all of whom were raised by their Japanese parents. All infants were full term at birth and were healthy at the time of the experiment. An additional 17 infants were excluded due to fussiness (*n* = 6) or failure to be familiarized to the speech movie (*n* = 11).

#### Stimuli and procedure

We used the same auditory stimuli and McGurk stimuli of the same two speakers, except for the discrepancy in the speaker’s identity of voice and face in the McGurk stimuli. We used Adobe Premiere Pro CS6 (Adobe Systems, San Jose, CA, USA) to manipulate the combination of face and voice in the McGurk stimuli for each race condition. For instance, in the own-race-face condition, we replaced the /pa/ voice of the own-race speaker with the /pa/ voice of the other-race speaker whose onset was adjusted at the same point as the original voice. As in Experiment 1, we set two conditions of speakers’ faces, the own-race-face (East Asian) and the other-race-face (Caucasian). Each condition consisted of a familiarization phase and a test phase. We assigned half of the infants in both age groups to the own-race-face condition, and the other half to the other-race-face condition. All other aspects of the apparatus, procedure, and data analysis were identical to Experiment 1. We also confirmed inter-observer reliability by calculating the correlation between the two observers’ ratios (*r* = .93).

### Results

#### Familiarization trials

Table 2 shows the mean total looking time across the first half and second half of familiarization trials in the own-race-face condition and the other-race-face condition. To confirm the decline of infants’ looking time during the familiarization phase in both conditions, we conducted a mixed ANOVA for each age group separately, with trials (the first-three and second-three trials) as a within-participants factor and the familiarization condition (the own-race-face and the other-race-face) as a between-participants factor. As in Experiment 1, the mixed ANOVA revealed a significant main effect for the trials in both 5–6-month-olds, *F*(1, 30) = 32.72, *p* < .01, η^2^ = .20, and 8–9-month-olds, *F*(1, 30) = 63.23, *p* < .01, η^2^ = .33. Neither the main effect of the stimulus nor an interaction effect was significant in 5–6-month-olds, main effect of stimulus, *F*(1, 30) = 1.71, *ns*; interaction, *F*(1, 30) = .13, *ns,* or in 8–9-month-olds, main effect of stimulus, *F*(1, 30) = 2.42, *ns*; interaction, *F*(1, 30) = 2.82, *ns*. These results indicate no difference in looking time during the familiarization phase between the own-race-face condition and the other-race-face condition in either age group.

**Table 2:**
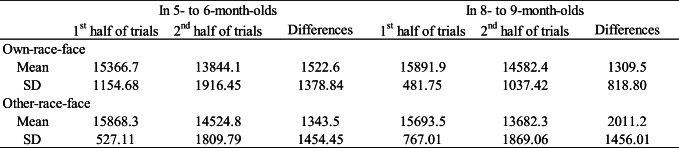
Mean total looking times (ms) across the first half and second half of the familiarization trials in both the own-race-face condition and the other-race-face condition

For the decreased looking time during the familiarization phase, we also calculated the difference in mean looking times between the first half and the second half of the familiarization trials and compared them between the two race conditions utilizing a two-sample *t* test. The *t* tests showed no significant difference in 5- to 6- month-old infants (*t* = .36, *df* = 30, *ns*) and 8- to 9-month-old infants (*t* = 1.68, *df* = 30, *ns*). To test the effect of face-voice combination of identity cues on familiarization, we conducted an ANOVA utilizing experiment (Experiments 1 and 2), condition (race), and number of months (age) as between-participants factors. The ANOVA showed no significant main effects for Experiment, *F*(1, 120) = .44, *ns,* as well as the Experiment × Race interaction, *F*(1, 120) = .03, *ns*. These results indicate that the loss of interest over the familiarization phase was similar to that in Experiment 2 and Experiment 1.

#### Test trials

**Fig. 3:**
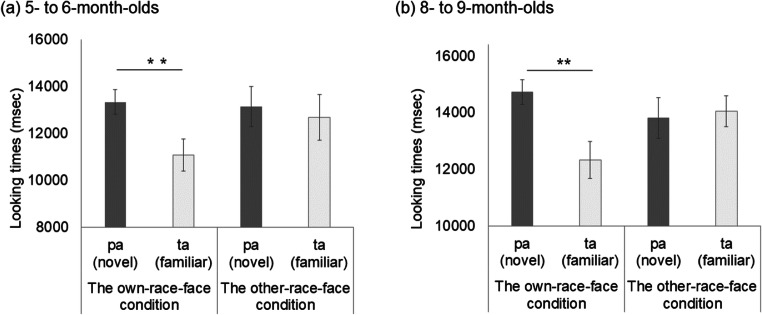
Mean total fixation times during the test phase of two familiarization conditions: in 5–6-month-olds (**a**) and in 8–9-month-olds (**b**). Dark-gray bars represent the results for the novel trials (the presentation of auditory /pa/ with vegetable images), and light-gray bars the results for the familiarized trials (the presentation of auditory /ta/ with vegetable images). The error bars represent ± 1 standard error of the mean. Asterisks indicate the significance level of the statistical differences: *p < .05

Figure 3 summarizes the mean total looking times during the test phase in both the own-race-face condition and other-race-face condition. As in Experiment 1, we conducted a mixed ANOVA separately for each age group, with trial as a within-participants factor and the familiarization condition as a between-participants factor. The mixed ANOVA showed that the main effect of stimulus was not significant in 5–6-month-olds, *F*(1, 30) = .00, *ns,* or in 8–9-month-olds, *F*(1, 30) = 1.55, *ns*, indicating no difference in total looking times during the test phase between the own-race-face condition and the other-race-face condition. Additionally, the mixed ANOVA revealed a significant Trials × Stimulus interaction in 8–9-month-olds, *F*(1, 30) = 10.18, *p* < .05, η^2^ = .07, although it was only marginally significantly in 5–6-month-olds, *F* (1, 30) = 2.90, *p* = .056, η^2^ = .05.

Next, we evaluated the novelty preference using multiple *t* tests (corrected using the Holm method) within each age group to compare the mean total looking time between the familiarized and novel trials in both race-face conditions. In 5–6-month-olds, the infants’ looking times were longer during the novel trial than during the familiarized trial in the own-race-face condition, *t*(15) = 2.85, *p* = .01, *d* = .91, while there was no difference of looking times between the novel and familiarized trial in the other-race-face condition, *t*(15) = .66, *ns*. A similar tendency was observed in 8–9-month-olds whereby infants’ looking times were longer during the novel trial than the familiarized trial in the own-race-face condition, *t*(15) = 4.71, *p* = .00, *d* = 1.08, but not in the other-race-face condition, *t*(15) = .37, *ns*. These results are consistent with the results of Experiment 1, indicating that infants showed a novelty preference for the /pa/ voice because they perceived the McGurk effect (/ta/) during the familiarization phase only in the own-race-face condition.

### Discussion

This experiment tested whether the results of Experiment 1 pertained to the other-race speaker’s accent rather than ethnic property of the face by manipulating the coherence of speech identity cues in the McGurk stimuli. Our results showed that the infants in both age groups could perceive the McGurk effect in the familiarization trials and then showed a novelty preference for /pa/ in the own-race-face condition but not the other-race-face condition. This replicated the results in Experiment 1 that infants from 5 months have the sensitivity to integrate auditory and visual speech only for the own-race-face speaker regardless of irrelevant speech identity cues.

Our results indicate that the other-race effect on the McGurk effect did not come from the effect of an unfamiliar accent in the audio stimuli. The results of Experiment 2 eliminated this possibility by showing that infants can perceive the McGurk effect when presented the own-race face with the other-race speaker’s voice, containing an unfamiliar accent. In addition, a further analysis also revealed that the amount of decreased looking time during the familiarization phase in Experiment 2 was the same as in Experiment 1. These results indicate that the infants have recognized the McGurk stimuli regardless of the difference between the own-race and the other-race speaker’s accent during familiarization. From these results, we believe that the unfamiliar accent presented in audio stimuli had no impact on perceiving the McGurk effect in the own-race-face condition.

Our results also indicate that, rather than intermodal relation with races and languages, facial information (i.e., race) has a significant impact on the McGurk effect in infants. Uttley et al. (2013) showed that infants around 6 months of age possess expectations about the sources of vocalizations based on their experiences, in that native language is associated with own-race faces and nonnative languages are associated with other-race faces. If such expectations influenced the results of Experiment 1, the other-race effect in the McGurk effect can be expected to disappear when the combination of race and language (i.e., face and voice) was flipped. Our results showed that the infants perceive the McGurk effect in the combination of the own-race face with the English voice, but not in the combination of the other-race face with the Japanese voice. This indicates that the infants ignored the discrepancy in the speaker’s identity cues and focused on the racial cue of the speaker’s face in audiovisual speech integration. Our results may suggest that a discrepancy in the speaker’s identity cues (face and voice) has essentially no impact on if infants can perceive multisensory coherence (e.g., de Boisferon et al., 2015; Patterson & Werker, 2002).

We further needed to confirm that infants in our two previous experiments were indeed showing a novelty preference for /pa/ because they experienced the McGurk effect (/ta/) during familiarization. One possibility is that the infants did not experience the McGurk effect and showed a familiarity preference for /pa/ voice in Experiments 1 and 2. To address this, we conducted Experiment 3 to examine whether the novelty preference was reversed when relevant facial information was removed from the familiarization (McGurk) stimuli. We considered the McGurk effect a result of integrating auditory and visual speech (e.g., Beauchamp et al., 2004; Nath & Beauchamp, 2012). In this study, the infants would perceive the McGurk effect in the own-race-face condition, because the novelty preference of /pa/ during test phase comes from the infants being familiar with the McGurk effect (/ta/). If relevant facial information was removed from the McGurk stimuli, such as the manipulation that voice (/pa/) was presented with the vegetable images, the novelty preference during the test phase would appear in reverse. Given a large body of evidence demonstrating that infants acquire such an ability from 1 month of age (e.g., Bertoncini & Mehler, 1981; Eimas et al., 1971; Jusczyk et al., 1978), the infants must be able to discriminate between the two auditory syllables (/pa/ and /ta/) regardless of speaker, even if auditory noise was added.

## Experiment 3

### Methods

#### Participants

Another 28 infants ages 5–6 months (17 girls and 11 boys; mean age = 173 days, range: 147 to 193 days) and 28 infants ages 8–9 months (17 girls and 11 boys; mean age = 258 days, range: 231 to 283 days) participated in Experiment 3, all of whom were raised by their Japanese parents. All infants were full term at birth and were healthy at the time of the experiment. An additional five infants were excluded due to fussiness (*n* = 2) or failure to be familiarized to the speech movie (*n* = 3).

#### Stimuli and procedure

We used four of the auditory stimuli used in Experiment 1 and set the own-race-face and the other-race-face conditions, where the name of each condition was inherited from the previous experiment. Each condition consisted of a familiarization phase and a test phase. The familiarization phase consisted of six trials, each of which consisted of the repeated presentation of the /pa/ voice with the same vegetable images as the test stimuli six times. The test phase consisted of two trials, a familiarized trial and a novel trial. In the familiarized trial, the voice “/pa/” was presented with images of vegetables six times. In the novel trial, the voice “/ta/” was presented with images of vegetables six times. We assigned half of the infants in both age groups to the own-race-face condition, and the other half to the other-race-face condition. All other aspects of the apparatus, procedure, and data analysis were identical to those of the previous experiments in this study. We also confirmed interobserver reliability by calculating the correlation between the two observers’ ratios (*r* = .94).

### Results

#### Familiarization trials

Table 3 shows the mean total looking time across the first half and second half of the familiarization trials in the own-race-face condition and the other-race-face condition. As in Experiment 2, we examined the decline of infants’ looking time during the familiarization phase by using a mixed ANOVA separately for each age group, with trials (the first-three and second-three trials) as a within-participants factor and the familiarization condition (the own-race-face and the other-race-face) as a between-participants factor. The mixed ANOVA revealed a significant main effect of the trials in both 5–6-month-olds, *F*(1, 26) = 91.63, *p* < .01, η^2^ = .35, and 8–9-month-olds, *F*(1, 26) = 51.83, *p* < .01, η^2^ = .36. Neither a main effect of the stimulus nor an interaction effect was significant in 5–6-month-olds, main effect of stimulus, *F*(1, 26) = 3.31, *ns*; interaction, *F*(1, 26) = .95, *ns,* or in 8–9-month-olds, main effect of stimulus, *F*(1, 26) = .01, *ns*; interaction, *F*(1, 26) = .04, *ns*. We also calculated the difference of mean looking times between the first half and the second half of the familiarization trials and compared them between the two race conditions using a two-sample *t* test. The *t* tests showed no significant difference in 5- to 6-month-olds infants (*t* = .97, *df* = 26, *.s*) and 8- to 9-month-olds infants (*t* = .20, *df* = 26, *ns*). These results indicate that there was no difference in looking time during the familiarization phase between the own-race-face condition and the other-race-face condition in either age group.

**Table 3:**
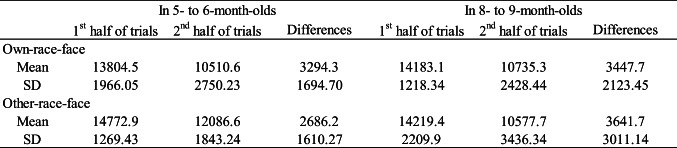
Mean total looking times (ms) across the first half and the second half of the familiarization trials in both the own-race-face condition and the other-race-face condition

#### Test trials

**Fig. 4:**
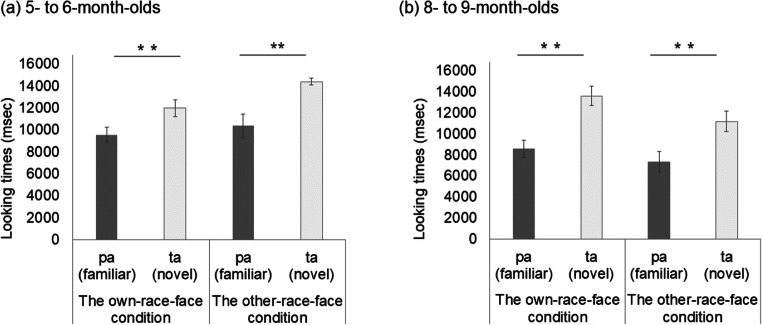
Mean total fixation times during the test phase of two familiarization conditions: in 5–6-month-olds (**a**) and in 8–9-month-olds (**b**). Dark-grey bars represent the results for the familiarized trials (the presentation of auditory /pa/ with vegetable images), and light-grey bars the novel trials (the presentation of auditory /ta/ with vegetable images). The error bars represent ± 1 standard error of the mean. Asterisks indicate the significance level of the statistical differences: *p < .05

Figure 4 summarizes the mean total looking times during the test phase in both the own-race-face condition and other-race-face condition. As in Experiment 2, we conducted a mixed ANOVA separately for each age group, with the trial as a within-participants factor and the familiarization condition as a between-participants factor. The mixed ANOVA analysis showed a significant main effect of the trial in 5–6-month-olds, *F*(1, 26) = 28.36, *p* < .01, η^2^ = .24, as well as in 8–9-month-olds, *F*(1, 26) = 55.33, *p* < .01, η^2^ = .29. Neither the main effect of the stimulus nor the interaction was significant in 5–6-month-olds, main effect of stimulus, *F*(1, 26) = 3.25, *ns*; interaction, *F*(1, 26) = 1.75, *ns,* or in 8–9-month-olds, main effect of stimulus, *F*(1, 26) = 2.37, *ns*; interaction, *F*(1, 26) = 1.02, *ns*.

To compare the mean total looking time between the familiarized and novel trials in both race-face conditions, we evaluated the novelty preference with multiple *t* tests (corrected using the Holm method) within each age group. In both age groups, the infants’ looking times were longer during the novel trial than the familiarized trial in both the own-race-face condition, in 5–6-month-olds, *t*(13) = 3.58, *p* = .003, *d* = .89; in 8–9-month-olds, *t*(13) = 5.81, *p* = .0001, *d* = 1.35, and the other-race-face condition, in 5–6-month-olds, *t*(13) = 4.01, *p* = .001, *d* = 1.54; in 8–9-month-olds, *t*(13) = 4.68, *p* = .0004, *d* = 1.04. These results indicate that, regardless of familiarization condition, infants showed a novelty preference for /ta/ voice when they were familiarized with /pa/ voice during familiarization.

### Discussion

The aim of this experiment was to confirm that infants in our two previous experiments were indeed showing a novelty preference for /pa/ because they have experienced the McGurk effect (/ta/) during familiarization. Therefore, we tested whether the novelty preference in Experiments 1 and 2 was reversed when relevant facial information was removed from the McGurk stimuli. Our results indicate that if infants in both age groups were familiarized with the /pa/ voice during the familiarization phase, they would show a novelty preference for /ta/ in the test phase in both familiarization conditions. These findings indicate that, the direction of the novelty preference flipped when facial information was removed, as we expected.

Our results support the results of our two previous experiments in that the novelty preference for /pa/ during the test phase came from the infants being familiarized with the McGurk effect (/ta/). Although the looking times during the familiarization phase in Experiment 3 were slightly less than those in other two experiments, such differences may be due to the difference in visual stimuli between experiments. That is, a facial stimulus used in Experiments 1 and 2 would attract more of an infant’s attention than a nonface object used in Experiment 3 (e.g., Di Giorgio et al., 2012), which may lead to the differences in looking during the familiarization period. However, this difference does not have a great impact on our results as the aim of this experiment was to confirm the basic ability to discriminate the two syllables. Taken together, these findings make it appear likely that infants can perceive the McGurk effect as a result of integrating auditory and visual speech in Experiment 1 as well as in Experiment 2. At the same time, it strongly suggests that infants from 5 months of age are likely to integrate auditory and visual speech information when observing own-race-face speech, but not other-race-face speech.

## General discussion

Through conducting three experiments, we demonstrated evidence that the other-race effect emerges in the development of audiovisual speech integration. In this study, we applied the McGurk effect (McGurk & MacDonald, 1976) to a familiarization/novelty preference paradigm in which it is assumed that infants show a novelty preference for /pa/ in the test phase if they can integrate voice and face, and then perceive the McGurk percept (/ta/) in the familiarization phase. We expected that the novelty preference for /pa/ would be found in the own-race-face condition but not in the other-race-face condition if the other-race effect has emerged. The results of Experiment 1 demonstrated that infants ages 5–9 months showed a novelty preference for /pa/ in the own-race-face condition but not in the other-race-face condition. In addition, the results of Experiment 2 confirmed that infants ages 5–9-months showed the same tendency as in Experiment 1 regardless of the difference between the own-race and the other-race speaker’s accent. Furthermore, the novelty preference was reversed when the relevant facial information was removed from the McGurk stimuli during the familiarization phase in Experiment 3.

Our data have implications for the hypothesis that perceptual narrowing is a modality-general, pan-sensory process (e.g., Lewkowicz & Ghazanfar, 2009; Pascalis et al., 2002; Pons et al., 2009). This hypothesis assumes the bidirectional effect of visual and auditory experiences such that audiovisual speech perception can be narrowed by increased experience with own-race faces as well as experience with one’s native language (Lewkowicz & Ghazanfar, 2009). The McGurk effect that we used in this study can assess the degree to which the infant can use a speaker’s face to perceive what the speaker says (McGurk & MacDonald, 1976; Rosenblum et al., 1997). Therefore, we believe that our results can provide evidence for this hypothesis with respect to the role of experience with own-race faces in the development of audiovisual speech perception. With respect to the role of experience with native languages, several studies have provided evidence; younger infants can match audiovisual speech in both native and nonnative languages, while older infants are no longer able to match audiovisual speech in nonnative languages because they no longer perceive a nonnative, auditory contrast (e.g., Kubicek et al., 2014a; Kubicek et al., 2014b; Pons et al., 2009). Given this bidirectional effect of visual and auditory experiences, it can be concluded that infants’ perceptual narrowing to specific faces and languages would gradually emerge as a modality-general, pan-sensory process during the second half of the first year of life (e.g., Lewkowicz & Ghazanfar, 2009; Pascalis et al., 2002; Pons et al., 2009).

We also found that the timing of the emergence of the other-race effect in the development of audiovisual speech perception was consistent with previous results in face perception (; Kelly et al., 2009; Xiao et al., 2018). Previous studies showed that the preference for own-race faces observed in 3-month-olds (Kelly et al., 2005) and the acuity for discriminating other-race faces decreased at around 6 months (Kelly et al., 2007). A recent study that recruited Japanese infants also reported the other-race effect in the face discrimination task at the same age (Xiao et al., 2018). In line with this, our results of the first two experiments also showed that infants aged 5- to 6-months can integrate voice with an own-race-face which they see it often, but not with an other-race-face that they rarely experience. These results indicate that the other-race effect is formed by the second half of the first year of life through experience with people of the child’s own race.

One important consideration of our findings is how the presence of the face affected integration—for example, whether it altered attention to the mouth, lip-reading ability, or the integration process. Previous studies showed that visual attention to the speaker’s mouth promoted the integration of audiovisual speech information (Altvater-Mackensen & Grossmann, 2016) and enhanced the perception of the McGurk effect (Gurler, Doyle, Walker, Magnotti, & Beaychamp, 2015). Thus, the other-race effect in our study may be a result of the difference in the visual attention to the mouth region between the own-race and other-race speakers’ faces. However, previous eye-tracking studies have provided insight into the similarity of infants’ scanning patterns of their own- and other-race faces (e.g., Liu et al., 2015; Minar & Lewkowicz, 2018; Singarajah et al., 2017). These studies have investigated the fixation distributions for some facial features (i.e., eyes, nose, mouth, and others), and found that infants’ attention to the mouth region did not differ between the own- and other-race faces when they viewed static faces (e.g., Liu et al., 2015; Singarajah et al., 2017). Additionally, a similar pattern was found when the infants viewed the audiovisual speech stimuli (e.g., Lewkowicz & Hansen-Tift, 2012; Minar & Lewkowicz, 2018). Given these results, we considered that infants in our study would have tended to pay attention to the mouth similarly in both race conditions, although we did not measure the gaze pattern in detail. However, our study did not rule out another possibility: that the results are due to differences in the ability to lip-read for the two faces. Indeed, previous studies showed that the lack of visual experiences during the early months following birth was related to worse performances in both lip-reading and audiovisual speech integration (e.g., Putzar, Goerendt, et al., 2010a; Putzar et al., 2007; Putzar, Hötting, et al., 2010b). Future studies should clarify what stage of processing (i.e., visual processing or integration) is affected by the presence of the face.

While the interpretation of our findings has been in the context of audiovisual integration, we should consider another interpretation focused on the infants’ cognitive resources for processing unimodal information (e.g., Reynolds & Romano, 2016; Xiao et al., 2018). From the framework of the familiarization paradigm, the familiarity preferences indicate incomplete encoding of the familiar stimulus during the familiarization phase, while the novelty preferences indicate full encoding of the familiar stimulus (e.g., Hunter et al., 1983; Hunter & Ames, 1988; Pascalis & de Haan, 2003; Rose et al., 1982). Considering just the auditory stimuli in our study, the infants were familiarized with auditory /pa/, then tested on auditory /pa/ versus /ta/. By assuming that the process for the static image of vegetable requires less cognitive resource than the process for the movie of facial speech, our results were interpreted as following: the infants showed a novelty preference for the /ta/ voice in Experiment 3 where the auditory stimulus was presented with vegetable images because they processed the familiarization stimuli (/pa/ voice). Whereas the infants showed a familiarity preference for the /pa/ voice in Experiments 1 and 2, where a moving face rather than vegetable accompanied the auditory stimulus, because they still continued to process the familiarization stimuli (/pa/ voice). This may indicate that the processing of facial stimuli distracted the infants’ cognitive resources, which leads to an impairment in the processing of the speech sound, rather than the processing of audiovisual integration. Indeed, the decrease of looking time during familiarization face was larger for Experiment 3 than for Experiments 1 and 2, which might imply the difference of the levels of achievement of familiarization. On the other hand, such explanation might not completely account for the other-race effect. Because, neither novelty nor familiarization preference in the test phase was found in the other-race condition in Experiment 1 and 2 even though there was no difference of looking times during familiarization phase between the own-race and other-race conditions. Given this, our results indicate that the racial difference in speaker’s face affect the processing of audiovisual speech stimuli, rather than an impairment in the processing of the speech sound.

Although previous research has demonstrated smaller McGurk effects in Japanese listeners compared with English listeners, these differences do not appear to emerge until 6–8 years (e.g., Sekiyama & Burnham, 2008; Sekiyama & Tohkura, 1991). Therefore, we believe that cultural differences had less impact on our results. In any case, the present study did demonstrate a McGurk effect, when infants saw an own-race face.

In summary, the current study demonstrated the other-race effect on the McGurk effect, indicating that infants are likely to integrate voice with an own-race face that they see often but not with an other-race face that they rarely experience. Our findings clarified the extended role of experience with faces in an infant’s development, which also supports the hypothesis that perceptual narrowing is a modality-general, pan-sensory process (e.g., Lewkowicz & Ghazanfar, 2009).

## Declarations

This experiment was conducted according to the Declaration of Helsinki and was approved by the Ethical Committee of Chuo University. Parents gave prior written informed consent for their children’s participation and for publication in an online open-access journal.
